# Genomic Characterization of the Guillain-Barre Syndrome-Associated *Campylobacter jejuni* ICDCCJ07001 Isolate

**DOI:** 10.1371/journal.pone.0015060

**Published:** 2010-11-29

**Authors:** Maojun Zhang, Lihua He, Qun Li, Honghe Sun, Yixin Gu, Yuanhai You, Fanliang Meng, Jianzhong Zhang

**Affiliations:** 1 Department of Communicable Disease Diagnostics (DCDD), Chinese Center for Disease Control and Prevention, National Institute for Communicable Disease Control and Prevention, Beijing, China; 2 Office for Disease Control and Emergency Response, Chinese Center for Disease Control and Prevention, Beijing, China; 3 Department of Applied Medical Science, Beijing Genomics Institute (BGI), Beijing, China; Charité - University Medicine Berlin, Germany

## Abstract

*Campylobacter jejuni* ICDCCJ07001 (HS:41, ST2993) was isolated from a Guillain-Barré syndrome (GBS) patient during a 36-case GBS outbreak triggered by *C. jejuni* infections in north China in 2007. Sequence analysis revealed that the ICDCCJ07001 genome consisted of 1,664,840 base pairs (bp) and one tetracycline resistance plasmid of 44,084 bp. The GC content was 59.29% and 1,579 and 37 CDSs were identified on the chromosome and plasmid, respectively. The ICDCCJ07001 genome was compared to *C. jejuni* subsp. *jejuni* strains 81-176, 81116, NCTC11168, RM1221 and *C. jejuni* subsp. *doylei* 269.97. The length and organization of ICDCCJ07001 was similar to that of NCTC11168, 81-176 and 81-116 except that CMLP1 had a reverse orientation in strain ICDCCJ07001. Comparative genomic analyses were also carried out between GBS-associated *C. jejuni* strains. Thirteen common genes were present in four GBS-associated strains and 9 genes mapped to the LOS cluster and the ICDCCJ07001_pTet (44 kb) plasmid was mosaic in structure. Thirty-seven predicted CDS in ICDCCJ07001_pTet were homologous to genes present in three virulence-associated plasmids in *Campylobacter*: 81-176_pTet, pCC31 and 81-176_pVir. Comparative analysis of virulence loci and virulence-associated genes indicated that the LOS biosynthesis loci of ICDCCJ07001 belonged to type A, previously reported to be associated with cases of GBS. The polysaccharide capsular biosynthesis (CPS) loci and the flagella modification (FM) loci of ICDCCJ07001 were similar to corresponding sequences of strain 260.94 of similar serotype as strain ICDCCJ07001. Other virulence-associated genes including *cadF*, *peb1*, *jlpA*, *cdt* and *ciaB* were conserved between the *C. jejuni* strains examined.

## Introduction

*Campylobacter jejuni* is a major food-borne pathogen and a major cause of human gastroenteritis world-wide. In addition to the burden of disease due to gastroenteritis, *C. jejuni* infections are significantly associated with the development of Guillain-Barré syndrome (GBS) [1].

In 2007, from the end of June to early July, an outbreak of 36 GBS cases was reported in Shuangyang, a township in Changchun, Jilin province, China. Serologic and bacterial studies indicated that infections with *C. jejuni* ICDCCJ07001 (07001) isolated from one severe GBS patient (who had been on machine ventilation for 180 days) were the cause of this outbreak,the largest GBS outbreak reported to date [2].

The Penner serotype and MLST type of this strain were HS: 41 and ST2993, respectively. In order to obtain the specific genetic characteristics of this *C. jejuni* 07001 isolate reported to trigger this GBS outbreak, the whole genome sequence was determined and comparative genomic analyses between the 07001 genome and previously sequenced *C. jejuni* strains was carried out.

## Materials and Methods

### Ethics Statement

Ethics approval for this study was obtained from the ethics committee in Chinese Center for Disease Control and Prevention (China CDC) and the academic committee in the National Institute for Communicable Disease Control and Prevention, China CDC. The verbal informed consent of the stool sample collection from the parents of the patient in this study during the outbreak was obtained and the data was analyzed anonymously (the verbal informed consent for stool sample collection is permitted in China CDC for emergency outbreak investigation). All the related documents were recorded in China CDC.

### Clinical characteristics associated with *C. jejuni* 07001 infection

*C. jejuni* 07001 was isolated from a stool specimen collected from a 15-year old female GBS patient that presented with diarrhea prior to the GBS outbreak [2]. The clinical subtype of this GBS case was classified as motor axonal neuropathy [AMAN] based on the clinical outcome, cerebrospinal fluid (CSF) examination and electromyogram (EGM) tests. Anti-*C. jejuni* antibodies (both IgM and IgG) were detected in serum and the dominant anti-ganglioside antibodies in serum were IgG anti-GM1 and IgG anti-GD1b. The serotype and MLST subtyping analysis demonstrated that the *C. jejuni* 07001 was HS:41 and ST2993, respectively [2].

### Bacterial culture and DNA extraction

*C. jejuni* 07001 was grown on Skirrow's selective medium (Columbia Agar Base, Oxoid CM0331, supplemented with 5% sheep blood and *Campylobacter* selective supplement, Oxoid SR0117) at 42°C under microaerobic conditions for 24 h. Total *C*. *jejuni* 07001 DNA was extracted using the DNeasy tissue kit (Qiagen, Valencia, CA) according to the manufacturer's protocol.

### Genome sequence

The 07001 genome was sequenced using the routine random shotgun method and the genomic libraries constructed and evaluated using standard procedures[3]–[5]. Briefly, sheared DNA samples were fractionated to construct two different genomic libraries containing average insert fragment sizes of 1.5–3.0 and 6.0–8.0 kb, respectively. The resulting pUC18-derived library plasmids were extracted using the alkaline lysis method and subjected to direct DNA sequencing with automated capillary DNA sequencers (MegaBACE1000, Piscataway, NJ) [5]–[6]. Assembly was performed using Phred-Phrap-Consed resulting in 9.4-fold genome coverage. PCR amplifications were utilized to close gaps. The genome and plasmid sequences were deposited in GenBank as CP002029 and CP002030, respectively.

### Gene annotations

Coding sequences (CDS) were predicted using GLIMMER2.0 and ORPHEUS. CDS were aligned using Exhaustive BLAST software against the whole *C. jejuni* 81-176 genome. The functions of respective CDS were annotated using BlastP (e-value <1e-10, 80% identity, and 100 amino acid overlap) and information derived from GenBank and UniProt (version 47). The functions of respective CDS were categorized using the Interpro, GO and Clusters of Orthologous Genes (COG) data bases. Finally, functional pathways were annotated based on KEGG pathway analyses. tRNA genes and repeats were predicted using tRNAscan-SE and Repeatmasker (http://repeatmasker.genome.washington.edu/cgi-bin/RMZ.pl)[5]–[6].

### Comparative genomics analysis

All nucleotide and predicted protein comparisons were performed using the BLAST package against the respective submitted *C. jejuni* genome updated nt and nr databases. Genomic comparisons were performed using BLASTN, BLASTX and Mummer at the whole-genome level. CDS homology was defined at both the DNA and amino acid levels by sequence similarities of greater that 80% and 35%, respectively. The completed genome sequences corresponding to *Campylobacter* strains *C. jejuni* subsp. *jejuni* 81-176, *C. jejuni* subsp. *jejuni* 81-116, *C. jejuni* subsp. *jejuni* NCTC11168, *C. jejuni* subsp. *jejuni* RM1221, *C. jejuni* subsp. *doylei* 269.97, *C. curvus* 525.92(NC_009715), *C. fetus* subsp. fetus 82-40(NC_008599), *C. hominis* BAA-381(NC_009714) and *C. concisus* 13826(NC_009802) were selected for the comparative genomic analysis against sequences corresponding to strain 07001. The comparative analyses were also performed between 07001 and three GBS-associated *C. jejuni* subsp. *jejuni* strains 260.94, HB93-13 and CF93-6 whose genomes are incompletely sequenced. Background information of the respective strains examined is described in the supplementary Table S1.

Six sequenced *Campylobacter* plasmids were selected for the comparative analysis (the supplementary Table S2). 81-176_pTet (RefSeq ID: NC_008790) and 81-176_pVir (RefSeq ID: NC_008770) were from a Campylobacteriosis outbreak-associated *C. jejuni* strain 81-176. HB93-13_pTet (RefSeq ID: NZ_AANQ01000006) is present in strain HS93-13, a GBS-associated *C. jejuni* strain from China. pCC31 (RefSeq ID: NC_006134) was from a *C. coli* strain isolated from a human case of severe gastroenteritis in the UK, pCCON31 (RefSeq ID: NC_009795) and pCCON16 (RefSeq ID: NC_009796) was present in a C. *concisus* isolate associated with a case of clinical gastrointestinal disease.

### NCBI blast and PCR screening for specific CDS in *C. jejuni* 07001

Unique *C. jejuni* strain 07001 CDS were identified using the NCBI nucleotide blast program: (http://blast.ncbi.nlm.nih.gov). The sequence of the entire unique *C. jejuni* 07001 CDS not present in the other *C. jejuni* isolates examined (*C. jejuni* 81-176, 81-116, NCTC11168, RM1221 and 269.97) were subjected to the NCBI blast database for the similar sequence analysis.

Since strain 07001 was the first GBS-associated *C. jejuni* isolate identified with a completely sequenced genome, characterization of genes unique to this strain may be useful in understanding mechanisms associated with disease presentation, in addition to providing novel targets for treatment modalities. Furthermore, comparative analysis of sequences between different *C. jejuni* isolates (including strain 07001) may provide evidence at the genetic level linking these GBS-related strains to disease presentation.

Thirty-three PCR-amplified 07001 CDS were compared to 61 *C. jejuni* strains isolated from north China including 6 outbreak-associated strains (3 from diarrheal patients and 3 from poultry), 6 strains isolated from patients presenting with GBS sporadically, 42 strains isolated from diarrheal patients and 7 strains from chicken fecal samples. Three pairs of primers were designed based on conserved regions from these genes and *C. jejuni* 07001 was used as the positive PCR amplification control.

## Results

### Genomic features of *C. jejuni* strain 07001

The *C. jejuni* 07001 genome was comprised of a chromosome and a tetracycline resistance plasmid. The genome was 1,664,840 bp long and predicted to contain 1,579 CDS. The 07001_pTet plasmid was similar to the 81-176_pTet plasmid which is 41,742 bp long and contains 52 CDS [7]. Additional *C. jejuni* 07001 genome characteristics are described in Table 1 and Table 2.

**Table 1:** Statistical analysis of the ICDCCJ07001 genome sequencing data.

Parameter	Value
Genomic library (1.5 kb∼3.0 kb)	1
Genomic library (6.0 kb∼8.0 kb)	1
No. of contigs	220
Total No. of reads	40,321
No. of reads assembled	34,008
Coverage depth (n-fold)	12
PCR reaction for gaps	336 (528 pairs primers)

**Table 2:** General genomic characteristics of *C. jejuni* ICDCCJ07001.

Characteristics	Genome	Plasmid
Length of sequence (bp)	1,664,840	44,084
G+C content	30.60%	28.69%
Repeat content length (%)	45,420 (2.73%)	1,753 (3.98%)
Simple repeats length (coverage)	504 (0.03%)	0 (0.00%)
Low complexity length (coverage)	38,568 (2.32%)	1,753 (3.98%)
Repeat element length	919	0
Small RNA #(length, coverage)	29 (5,429,0.33)	0 (0,0.00%)
ORF (Length in Genome)	1,498,305	35,802
Feature		
ORF #	1,579	37
Genome coverage (%)	90.00%	81.21%
Genome density (genes/Kb)	0.95	0.84
Average gene length (bp)	949	968
Max gene length (bp)	4,554	5,808
Function		
ORF with assigned functions	1,546	29
Conserved hypothetical protein	30	1
ORF without database match	3	7
RNA		
rRNA	3x(16S-23S-5S)	0
tRNA	44	0
Clusters of Orthologous Group(COG)-ORF#	1,519	15
KEGG-	1,560	19
Swissprot	1,064	11

### Comparative analysis of different *C. jejuni* genomes

**Figure 1:**
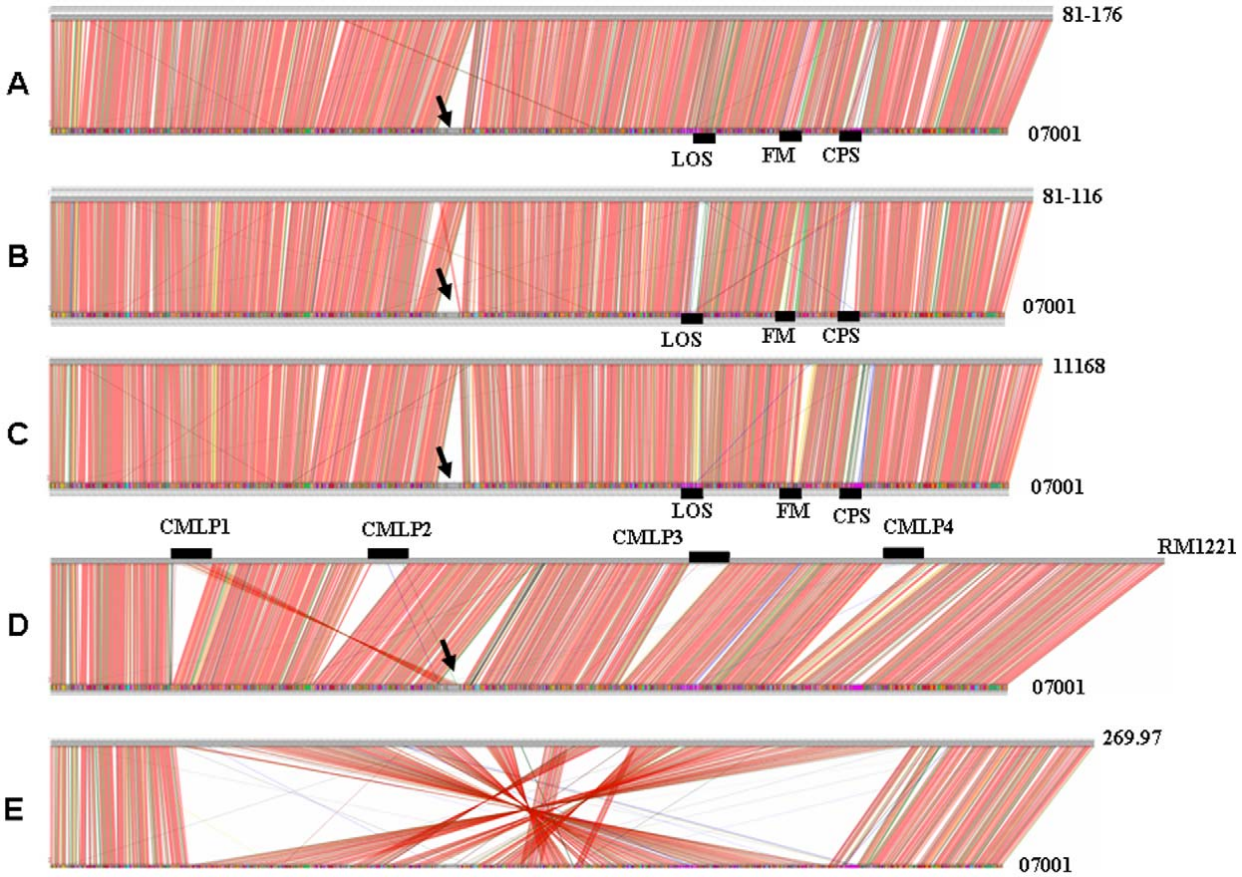
Linear genome comparisons between *C. jejuni* subsp. *jejuni* 07001, *C. jejuni* 81-176, 81-116, NCTC11168, RM1221 and *C. jejuni* subsp. *doylei* 269.97. BLASTN analyses were performed between *C. jejuni* 07001 and five other sequenced genomes of strains: *C. jejuni* subsp. *jejuni* 81-176 (A), 81-116 (B), NCTC11168 (C), RM1221 (D) and *C. jejuni* subsp. *doylei* 269.97 (E). The length and organization of the 07001 isolate were similar to those described for NCTC11168, 81-176 and 81-116 except for the reverse insertion of CMLP1 found in strain RM1221. Genome rearrangements were found between *C. jejuni* subsp. *jejuni* 07001 and *C. jejuni* subsp. *doylei* 269.97. Specific CDS in *C. jejuni* subsp. *jejuni* 07001 are listed in Table 3 and the homologous CDS between *C. jejuni* subsp. *jejuni* 07001 and 81-176, 81-116, NCTC11168, RM1221 and *C. jejuni* subsp. *doylei* 269.97 are summarized in the supplementary materials. Colors of the lines represent the identity of the DNA sequence between different CDS. Red, 90%–100%; green, 80%–89%; gold, 60%–79%; brown, 50%–59% and blue, 35%–49%. The arrow indicates the region of the reversed CMLP1 on the *C. jejeuni* 07001 chromosome.

*C. jejuni* genome alignments were carried out using Blastn. The greatest region of variability between strains 07001, 81-176, 81116 and NCTC11168 was observed between positions 668,019-7,07,030 (from CDS 07001_659 to CDS 07001_699; 39 kb) which contains part of the *Campylobacte*r Mu-like phage 1(CMLP1) in strain 07001. Three major highly divergent regions ranging between positions 1,107,958–112,069 (18 kb), 1,269,226–1,295,548 (26 kb) and 1,375,259–1,412,737 (37 kb) encoding for LOS, FM and CPS loci, respectively, were detected between these four human *C. jejuni* subsp. *jejuni* strains (Fig. 1A–C). The genome of the *C. jejuni* chicken isolate RM1221 was significantly larger than that of strain 07001. Three genomic islands located between positions 485,863–538,756 referred to as *C. jejuni* integrated elements (CJIE2, 52 kb), 1,021,099–1,071,856 (CJIE3, 50 kb), 1,335,719–1,371,944 (CJIE4, 36 kb) in RM1221 were absent from strain 07001. A region pertaining to the first genomic island, CJIE1 (also termed CMLP1) in strain RM1221 was present in reverse orientation compared to strain 07001 (between positions 68,019–707,030; 39 kb) (Fig. 1D). Chromosome rearrangement between positions 2,39,789–1,361,310 in strain 07001 (1.12 Mb) was observed resulting in 67% of the 07001 genome and that of *C. jejuni* subsp. *doleiy* strain 269.97 (Fig. 1E). The highest level of variability between the studied *C. jejuni* isolates was observed in the regions associated with the biosynthesis of surface structures such as flagellin, LOS, CPS, FM, metabolic, biosynthestic and regulatory processes and the DNA restriction modification (R/M) locus. The sketch maps of the comparative results are described in Fig. 1.

### Homopolymeric G_C tract analysis

A total of 25 poly (G_C) tracts were found in strain 07001, similar to other *C. jejuni* strains (29 in NCTC11168, 25 in RM1221 and 19 in 81–176) [8]–[10]. The location of the poly (G_C) tracts is described in the supplementary Table S3. Eighteen tracts were found in the CDS region and 7 in the intergenic region. Among the 25 tracts detected in *C. jejuni* strain 07001, 6 were found in the CPS region, 6 in the FM region, 1 in the type II restriction-modification enzyme region and 3 tracts in the intergenic region between genes located on the variable region on the genome.

### CDS homology between C. *jejuni* isolates

CDS sequences of the reference strains were obtained from NCBI according to the RefSeq ID. DNA and predicted protein sequences were blasted with the corresponding sequenced genes and predicted protein sequences corresponding to strains 81-176, 81-116, NCTC11168, RM1221 and 269.97 using Blast with default parameters. 1,179 homologous genes from strain 07001 were also found in the genomes of the 6 *C. jejuni* strains examined in this study. 1,351 homologous genes from strain 07001 were also observed in the human 81-176, 81-116 and NCTC11168 isolates. There were 1,441, 1,416, 1,396, 1,409 and 1,229 homologous genes between strains 07001 and 81-176, 81-116, NCTC11168, RM1221, and 269.97, respectively. 138, 163, 183, 170 and 350 CDS present in 07001 were absent from strains 81-176, 81-116, NCTC11168, RM1221, and 269.97. However, 236, 256, 284, 468, and 544 CDS were not observed in strain 07001 that were specific to strain 81-176, 81-116, NCTC11168, RM1221 and 269.97. 1,316 homologous genes were common between *C. jejuni* subsp. *jejuni* strains 81-176, 81-116, NCTC11168 and RM1221 and 137 genes were absent from the *C. jejuni* subsp. *doylei* 269.97 genome. Thirty-two genes were unique to strain 07001, specifically CDS present in strain 07001 absent from 81-176, 81-116, NCTC11168, RM1221 and 269.97 were listed in Table 3. The homologous CDSs between *C. jejuni* subsp. *jejuni* 07001 and 81-176, 81-116, NCTC11168, RM1221 and 269.97 and the strain specific CDSs were summarized in the Supplementary File S1, File S2, File S3, File S4.

**Table 3:** 32 Specific CDS from *C. jejuni* ICDCCJ07001.

CDS Names	CDS Description
ICDCCJ07001_132	McrBC restriction endonuclease system
ICDCCJ07001_766	hypothetical protein
ICDCCJ07001_824	FspA2 (fspA2)
ICDCCJ07001_1095	putative glycosyl transferase (LOS)
ICDCCJ07001_1096	beta-1,4-N-acetylgalactosaminyltransferase (LOS)
ICDCCJ07001_1102	acetyltransferase (LOS)
ICDCCJ07001_1103	Acetyltransferase (LOS)
ICDCCJ07001_1346	putative Na+/H+ antiporter (CPS)
ICDCCJ07001_1347	hypothetical protein (CPS)
ICDCCJ07001_1348	Pyruvate kinase, barrel domain (CPS)
ICDCCJ07001_1349	putative sulfate adenylyltransferase (CPS)
ICDCCJ07001_1351	putative glycosyltransferase (CPS)
ICDCCJ07001_1352	putative sugar transferase (CPS)
ICDCCJ07001_1355	putative sugar transferase (CPS)
ICDCCJ07001_1361	nucleotidyl-sugar pyranose mutase (CPS)
ICDCCJ07001_1363	UDP-galactopyranose mutase (CPS)
ICDCCJ07001_1364	WbcB (CPS)
ICDCCJ07001_1365	dTDP-4-dehydrorhamnose 3,5-epimerase (CPS)
ICDCCJ07001_1366	CDP-glucose 4,6-dehydratase (CPS)
ICDCCJ07001_1367	glucose-1-phosphate cytidylyltransferase (CPS)
ICDCCJ07001_1368	putative sugar transferase (CPS)
ICDCCJ07001_659	conserved hypothetical protein (CMLP1)
ICDCCJ07001_693	hypothetical protein (CMLP1)
ICDCCJ07001_699	hypothetical protein (CMLP1)
ICDCCJ07001_78	transcriptional regulator, Crp/Fnr family protein
ICDCCJ07001_935	putative periplasmic protein
ICDCCJ07001_1468	Streptomycin resistance protein (Oxidative phosphorylation region)
ICDCCJ07001_1469	helicase, putative (Oxidative phosphorylation region)
ICDCCJ07001_1470	helicase, putative (Oxidative phosphorylation region)
ICDCCJ07001_728	ATP-dependent endonuclease (type I restriction-modification system)
ICDCCJ07001_729	ATP/GTP-binding protein (type I restriction-modification system)
ICDCCJ07001_730	ATP/GTP-binding protein (type I restriction-modification system)

### Comparative analysis of the virulence locus between *C. jejuni* isolates

Fifteen LOS loci CDS were predicted for strain 07001. The organization and the cluster sequence showed high degrees of similarity with *C. jejuni* strains HB93-13, CF93-6 and 260.94 (GBS-associated strains) and based on the sequence similarity with *C. jejuni* strains ATCC43432 (AF215659) and ATCC43438 (AF400048) reported to have a LOS type similar to other GBS-associated strains, classified as type A [11]–[12]. In addition, the virulence genes *cgtA*, *cgtB* and CstII were found in the 07001 genome.

CPS is another *C. jejuni* cell surface-associated glycolipid reported to have no chemical links to LOS but demonstrated to be a critical component of the Penner serotype recently described[13]. A gene cluster was identified to be involved in CPS biosynthesis in *C. jejun*i (from cj1414-cj1443 in strain NCTC11168) that contained a central variable biosynthetic region flanked by two sets of conserved *kps* genes with high similarity to genes involved in transport and assembly of CPS in *E. coli* and other bacteria[13]–[14]. The comparative results of the CPS cluster between 07001 and other *C. jejuni* strains showed that it was a highly variable region. Thirty-one genes from the 07001 genome were between positions 1346–1376 (cj1415c–cj1442c).

There were 6, 4, 4, 3 and 5 homologous genes between strain 07001 and *C. jejuni* 81-176, 81-116, NCTC11168, RM1221 and 269.97. 5 and no homologous genes observed in the CPS cluster between strain 07001 and two other GBS-associated strains CF93-6 and HB93-13. However, strains 07001and 260.94 of the same serotype had CPS cluster genes that were 100% similar.

The FM cluster has been shown to play an important role in antigenic specificity and expression of flagellar structures in *Campylobacter* species. Twenty-nine genes were defined for strain 07001 and as observed for strains NCTC11168 and 81-176, genes in this cluster were highly variable between the selected *C. jejuni* strains examined [8].

Twenty-seven virulence and virulence associated genes common to Campyl*obacter sp*. were compared between the selected *C. jejuni* strains. Three CDT genes (*cdtA*, *cdtB and cdtC*) and two sensor histidine kinase genes (ICDCCJ07001_1169, ICDCCJ07001_1419) were missing from *C. jejuni* subsp. *doylei* 269.97 but the remaining 22 genes were conserved in the *C. jejuni* subsp. *jejuni* strains 07001, 81-176, 81116, NCTC11168 and RM1221. The comparisons of the virulence and virulence associated genes were listed in the supplementary File S5.

### Common CDS sequences between pathogenic *C. jejuni* strains associated with outbreaks of human disease

Various *C. jejuni* isolates associated with disease outbreaks in humans were used in the comparative genomic analysis with the GBS 07001 strain genome. Specifically, *C. jejuni* 81-176 was originally isolated from a diarrheal outbreak associated with raw-milk consumption in 1985 and has been shown to cause inflammatory colitis in two human infection challenge studies [15]–[16], *C. jejuni* 81-116 was originally isolated from a water-borne human outbreak resulting in gastroenteritis [17] and *C. jejuni* NCTC11168 was originally isolated from a case of human enteritis and was the first *C. jejuni* genome sequenced [9]. 1,476, 1,495 and 1,485 genes, respectively, were re-defined for strains 81-176, 81-116 and NCTC11168 according to the same strategy used to describe strain 07001 at the genomic level (the length of each CDS sequence >300 bp). 1,380 genes were common (homologous) between strains 07001, 81-176 and 81-116 and there were 1,384 homologous genes between strains 07001 and NCTC11168.

Strain 260.94 (HS:41, ST-362, ST-362 complex) was isolated from a patient presenting with severe GBS identified at the Red Cross Children's Hospital in Cape Town, South Africa [18]. The serotype and ST complex of this isolate was HS:41 and ST-362, respectively, similar to what was described for strain 07001. 1,538 ORFs were predicted for the Cape Town isolate based on analysis of the uncompleted genome sequence avaialble from NCBI (contig-RefSeq ID: NZ_AANK01000007, NZ_AANK01000006, NZ_AANK01000005, NZ_AANK01000004, NZ_AANK01000003, NZ_AANK01000002, NZ_AANK01000001, NZ_AANK00000000). From the blast results, 1,570 (99%) CDS from strain 07001 were homologous to strain 260.94. Only 9 CDSs which encoded 2 iron-binding proteins (07001_67, 07001_68), 2 hypothetical proteins (07001_37, 07001_134), 2 type II restriction-modification enzymes (07001_36, 07001_1013), 2 endo-nucleases (07001_132, 07001_133) and 1 lipo-oligosaccharide biosynthesis glycosyltransferase (07001_1106) were unique to *C. jejuni* 07001.

HB93-13 (HS:19) and CF93-6 (serotype unknown) are both GBS-associated *C. jejuni* subsp. *jejuni* strains isolated in Asia [19]. HB93-13 is a well characterized strain isolated from the feces of an eight-year-old boy in China that was diagnosed with the acute motor axonal neuropathy (AMAN) resuling from GBS [20]. CF93-6 was isolated from a patient diagnosed with MFS in Japan [21]. 1,550 and 1,445 CDS (CDS >300 bp) were predicted in CF93-6 and HB93-13 according to reference sequences NZ_AANQ00000000 and NZ_AANJ00000000, respectively. 1,383 homologous genes were common between 07001, HB93-13 and CF93-6.142 and 134 CDS present in strain 07001 were missing from both HB93-13 and CF93-6 genome sequences. Most of them encoded regions in CPS and FM. CMLP1was also absent in strain HB93-13.

**Figure 2:**
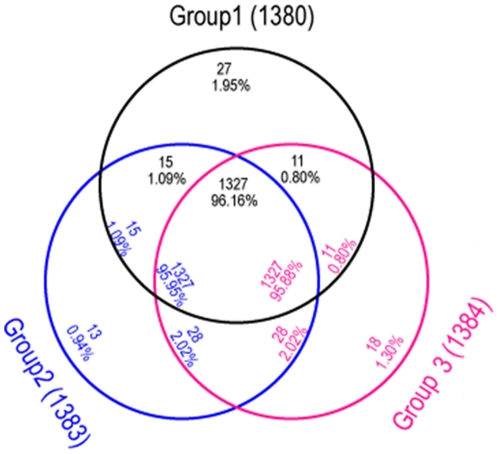
Venn diagram and in silico comparison between three *C. jejuni* strains. Group 1 (Campylobacteriosis outbreak strains *C. jejuni* 81-176 and 81-116 shared 1,380 homologous genes with strain 07001, 81-176 and 81-116. Group 2 (GBS associated strains including *C. jejuni* 260.94, HB93-13, and CF93-6) shared 1,383 homologous genes with *C. jejuni* 07001, 260.94, HB93-13 and CF93-6. Group 3 (enteritis associated strains including *C. jejuni* NCTC11168) shared 1,384 homologous genes with strains 07001 and NCTC11168. 1,327 homologous genes were common between all groups. 27, 13 and 18 CDS were specific to the Campylobacteriosis outbreak-associated group, GBS-associated strains and enteritis strains, respectively.

*C. jejuni* isolates were classified in the context of disease presentation: Group 1 strains (strains 81-176 and 81-116) were identified during the course of a Campylobacteriosis outbreak episode, Group 2 strains consisted of GBS-associated organisms (strains 260.94, HB93-13 and CF93-6) and Group 3 (strain NCTC11168) consisted of an enteritis-related isolate. There were 1,380, 1,383 and 1,384 homologous genes identified between strain 07001 and group 1 strains, group 2 strains and group 3 strains, respectively. Among these genes, 1,327 were common to all groups compared to 27 and 13 unique CDS identified between the Campylobacteriosis outbreak-associated and GBS-associated groups, respectively. Of the 13 GBS-associated genes, 9 mapped to the LOS loci. The comparative results based on group classification are described in Fig. 2 and CDS corresponding to the different groups are listed in the supplementary Table S4.

### Plasmid comparison between *C. jejuni* isolates

**Figure 3:**
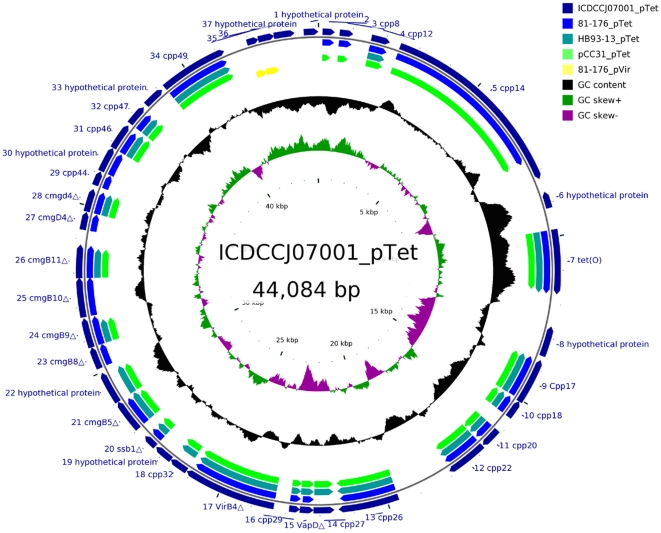
Homologous CDS among six Campylobacter plasmids. Circular plots demonstrating the mosaic structure of the 07001_pTet plasmid and homologous CDS between the 07001_pTet and other *Campylobacter* plasmids. The tracks from the outside represent: (1) the entire 37 CDS on plasmid 07001 (dark blue), (2) homologous CDS on plasmid 81-176_pTet (blue), (3) homologous CDS on plasmid HB93-13_pTet (dark green), (4) homologous CDS on plasmid PCC31_pTet (green), and (5) 81-176_pVir (yellow). 26, 21, 24 and 2 homologous CDS were identified between 07001_pTet and 81-176_pTet, HB93-13_pTet, PCC31_pTet and 81-176_pVir.

Thirty-seven predicted CDS were identified in the 07001_pTet plasmid that were homologous to various genes present in the four virulence associated plasmids from *Campylobacter* species including 81-176_pTet (26 homologous genes), pCC31 (24 homologous genes), HB93-13_pTet (21 homologous genes) and 81-176_pVir (2 homologous genes). Five CDS (07001_pTet000001, 07001_pTet000006, 07001_pTet000008, 07001_pTet000033 and 07001_pTet000037) were unique to 07001_pTet. No homologous genes were found between 07001_pTet and pCCON31 or pCCON16. Ten of 37 genes from 07001_pTet were homologous to type IV secretion system (T4SS) proteins which were also found in 81-176_pTet and pCC31 [7]. The *repA* determinant, however, was absent from 07001_pTet. The mosaic structure of the 07001_pTet plasmid and the homologous CDS are descried in Fig. 3.

### NCBI blast and PCR screening results

NCBI online BLAST program analysis identified 8/32 CDS specific to strain 07001 following comparisons to other *C. jejuni* strains and 10/27 CDS were unique to strains associated with Campylobacteriosis outbreaks. Thirty-three CDS, including 8 CDS unique to strain 07001 consisted of 3 conserved CDS in the CMLP1 region, 10 common CDS associated with the Campylobacteriosis out-break-associated *C. jejuni* strains 81-176 and 81-116, 8 CDS from plasmid 07001_pTet (07001_ pTet) and 4 common CDS located outside of the LOS region identified among the 4 GBS-associated *C. jejuni* isolates were selected as PCR screening targets. All 33 targets were identified in three human GBS outbreak-associated isolates. However, only 7, 5 and 4 CDS were positive in 3 chicken isolates examined, respectively.

**Figure 4:**
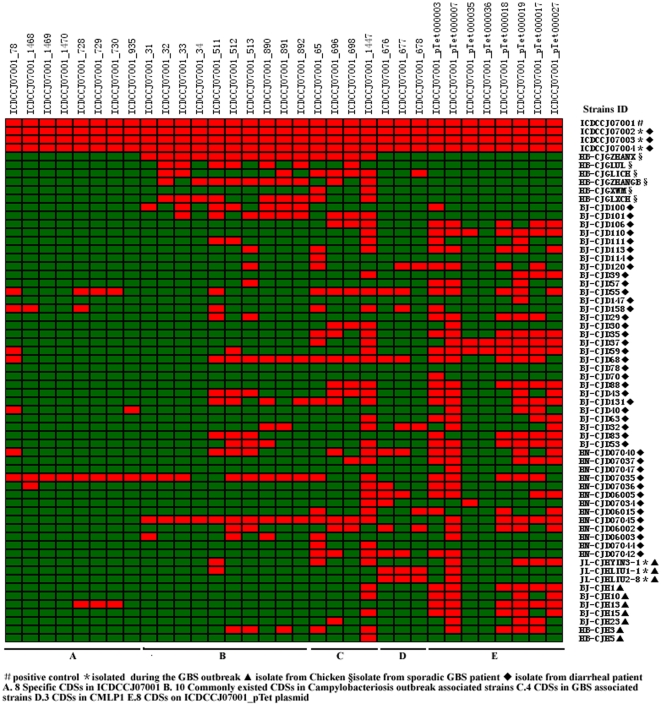
PCR screening results of 33 CDS for 61 *C. jejuni* isolates in China. 33 selected CDS were screened by PCR using three pairs of primers designed to amplify conserved regions present in the 61 Chinese isolates. *C. jejuni* 07001 was taken as the positive control for PCR amplification. The red and green bars indicate positive and negative results, respectively.

All three poultry isolates were negative for the eight specific *C. jejuni* 07001 CDS. Except for the outbreak-associated human isolates, 46/58 (79%) isolates were positive for the 4 GBS-associated CDS, 29/58 (50%) isolates were positive for 10 Campylobacteriosis-associated CDS, 11/58 (19%) isolates were negative for all 8 plasmid CDS and 39/58 (67%) were positive for 07001_pTet_000007. Forty-three (43/58, 74%) isolates were negative for all three CMLP1 CDS. PCR screening results of 33 CDS for 61 *C. jejuni* isolates were presented in Fig. 4.

## Discussion

*C. jejuni* subsp. *jejuni* strains 81-176, 81-116, NCTC11168 and *C. jejuni* subsp. *dolyei* 269.97 were isolated from infections of humans and *C. jejuni* subsp. *jejuni* RM1221 was isolated from the skin of an infected chicken [9], [22]–[24]. Except for the reverse orientation of CMLP1, the genomic content and chromosomal structure of *C. jejuni* 07001 were similar to other human *C. jejuni* subsp. *jejuni* isolates. The hypervariable regions present in the human isolates present in the portions of the genome associated with the synthesis and modification of cell surface structures were also similar to previous reports [8]. The RM1221 genome was significantly larger than the other 4 human isolates examined as a consequence of 4 CJIE insertions. CMLP1 is one of the prophages associated with *C. jejuni* isolates and reported to be common to most *C. jejuni* strains [10], [25]. Homologous CMLP1 sequences were also observed in isolates associated with a waterborne outbreak of gastroenteritis in Walkerton, Canada [26]. Although a defined role for CMLP1 has not been established in the context of virulence, it role in *C. jejuni* development has been previously described [25]–[26]. In this study, PCR analyses of the three conserved CMLP1 genes identified that 5/6 GBS outbreak-associated isolates were positive for all 3 CMLP1 genes. One or 2 genes were present in 13/55 (24%) isolates screened suggesting a heterogeneous distribution of CMLP1 among *C. jejuni* isolates in China. Extensive genetic chromosomal rearrangements were observed between *C. jejuni* subsp. *jejuni* and *C. jejuni* subsp. *dolyei* 269.97. However, 78% (1229/1579) homologous CDS were observed in this study between strains 07001 and 269.97. The mechanism and the role of the observed chromosomal rearrangements between these two sub-species was unclear, however, these changes may be associated with changes in *C. jejuni* virulence.

Variable poly (G_C) tracts in *C. jejuni* were first reported in the genome sequence of strain NCTC11168 [9]. Subsequent studies indicated that variable poly (G_C) tracts in *C. jejuni* contributed to pathogen heterogeneity [22], [27] and the high levels of phase variation between surface antigens was a result of slipped-strand misspairing [14], [28]–[29]. In this study, the highest levels of G_C heterogeneity were observed in the regions of greatest variability. Although not supported by experimental evidence, these observations suggested that the variability in G_C tracts may be responsible for phase-variation between *C. jejuni* isolates.

A significant number of homologous CDS (1351/1579, 86%) were consistently present in the 4 human isolates examined: *C. jejuni* subsp. *jejuni* 07001, 81-176, 81-116 and NCTC11168 and 83% (1316/1579) of homologous CDS were observed in 5 *C. jejuni* subsp. *jejuni* 07001, 81-176, 81-116, NCTC11168 and RM1221 and 75% (1179/1579) of homologous CDS were present among 7 *C. jejuni* isolates (07001, 81-176, 81-116, NCTC11168, RM1221 and 269.97). Most of the conserved virulence-associated factors identified between the 6 *C. jejuni* isolates were consistent with previously reported data [30]–[31].

Comparative analysis of the respective genomes identified 32 CDS unique to strain 07001 including three predicted proteins, ICDCCJ07001_132 (McrB subunit), ICDCCJ07001_766 (hypothetical protein), ICDCCJ07001_824 (FspA2). FspA2 is a *C. jejuni* flagellum-secreted protein reported to be a virulence factor associated with the induction of epithelial cell apoptosis [32], however, the function of *fspA2* in 07001 remains undefined. Fourteen of the 07001-specific genes mapped to the CPS biosythesis loci, 4 to the LOS biosynthesis loci and 3 to CMLP1. Eight unique 07001 genes were identified following NCBI blast analysis. Three unique CDS belonged to the type I restriction-modification system, 3 to the oxidative phosphorylation pathway and 2 as a putative periplasmic protein and a Crp/Fnr family protein, respectively. The PCR screen demonstrated that only 9/57 tested isolates were positive for 1 or 8 CDS except for 3 GBS outbreak-associated isolates. One isolate (HN-CJD07035) from a diarrheal patient was positive for all 8 CDS. Further analysis used to better define molecular differences between 07001 and HN-CJD07035 will have to be carried out to more clearly define critical virulence determinants.

The gene content and organization of the LOS biosynthesis locus in *C. jejuni* is considered critical for disease caused by respective isolates to present as GBS [33]–[36]. The LOS biosynthesis region exhibited significant variation between the strains examined. Based on gene content and organization, the loci were divided into 11 classes (A–L) [35]. The class A locus was reported to be predominately associated with GBS-associated isolates as previously described [37]–[38]. According to sequence comparisons, the organization and the 07001 LOS sequence was highly similar to the sequence/organization of *C. jejuni* strains HB93-13, CF93-6 and 260.94 also part of the class A locus. The specific gene content of the LOS loci might result in differences in biosynthetic pathways. Furthermore, the simultaneous presence of the *cgt*A, *cgt*B and c*st*-II genes in the LOS loci were significantly associated with GBS-associated *C. jejuni* isolates [39].

In this study, the *C. jejuni* isolates studies were clustered into 3 groups based on their respective genomic sequences. Thirteen CDS were identified from GBS-associated isolates and interestingly, 9 belonged to the LOS biosynthesis loci supporting previous observations [33]–[39]. An additional 4 CDS were also associated with the 4 GBS-associated strains studied. PCR screen results showed that 46/58 tested isolates were positive for 1 or 4 of these genes. Among the GBS isolates associated with sporadic disease episodes, 4 genes were present in 2/6 strains. Two genes were present in 3 isolates and 1 present in the remaining isolate. There was no difference in the distribution of these genes between GBS-associated isolates and other *C. jejuni* isolates suggesting that these genes were likely distributed across many *C. jejuni* strains.

Recent biochemical and genetic studies have shown that CPS in *C. jejuni* defines the Penner serotype [13]–[14]. The comparative analysis carried out in this study identified high degrees of variability within the CPS gene cluster and in strain 07001 this cluster was significantly similar to that defined in strain 260.94 but different from sequences described for other GBS-associated isolates. These results confirmed that it is the CPS and not LOS loci associated with the *C. jejuni* Penner serotype.

Ten CDS were found unique to isolates associated with a Campylobacteriosis outbreak following comparative analysis and NCBI blast sequence comparisons. PCR results showed that 29/58 (50%) of the tested isolates were positive for 1–10 genes. Two isolates, including one isolate from a patient presenting with sporadic GBS was positive for all of the 10 genes. The function of these genes in the context of pathogenesis and disease presentation needs to be further examined.

The three GBS outbreak-associated human isolates contained similar CDS different from CDS present in poultry isolates, suggesting that poultry species are not acting as reservoir hosts resulting in human infections, consistent with previous bacterial subtyping analysis [2].

This study profiled specific genetic characteristics associated with the *C. jejuni* 07001 isolate. However, further studies will be needed to define the specific nature of virulence factor pathogenesis pathways associated with epidemic infections as a means of better understanding mechanisms of disease associated with GBS-associated *C. jejuni* isolates.

## Supporting Information

File S1 **1,179 Commonly presented homology CDSs in** ***C. jejuni*** **subsp.** ***jejuni*** **07001,** ***C. jejuni*** **81-176, 81-116, NCTC11168, RM1221 and** ***C. jejuni*** **subsp.** ***doylei*** **269.97.** BLASTN analyses were performed between *C. jejuni* 07001 and five other sequenced genomes of strains: *C. jejuni* subsp. *jejuni* 81-176, 81-116, NCTC11168, RM1221 and *C. jejuni* subsp. *doylei* 269.97. 1,179 homologous genes from strain 07001 were also found in the genomes of the 6 *C. jejuni* strains examined in this study. The homologous CDSs were summarized in the Supplementary File S1.
(XLS)

File S2 **1,351 Commonly presented homology CDSs in the human** ***C. jejuni*** **subsp.** ***jejuni*** **07001, 81-176, 81-116 and NCTC11168 isolates.** BLASTN analyses were performed between *C. jejuni* 07001 and three other sequenced genomes of human *C. jejuni* subsp. *jejuni* 81-176, 81-116 and NCTC11168 isolates. 1,351 homologous genes from strain 07001 were also observed in the human 81-176, 81-116 and NCTC11168 isolates. The homologous CDSs were summarized in the Supplementary File S2.
(XLS)

File S3 **Homology CDSs between** ***C. jejuni*** **subsp.** ***jejuni*** **07001,** ***C. jejuni*** **81-176, 81-116, NCTC11168, RM1221 and** ***C. jejuni*** **subsp.** ***doylei*** **269.97.**
BLASTN analyses were performed between *C. jejuni* 07001 and five other sequenced genomes of strains: *C. jejuni* subsp. *jejuni* 81-176, 81-116, NCTC11168, RM1221 and *C. jejuni* subsp. *doylei* 269.97. There were 1,441, 1,416, 1,396, 1,409 and 1,229 homologous genes between strains 07001 and 81-176, 81-116, NCTC11168, RM1221, and 269.97, respectively. The homologous CDSs between *C. jejuni* subsp. *jejuni* 07001 and 81-176, 81-116, NCTC11168, RM1221 and 269.97 were summarized in the Supplementary File S3.
(XLS)

File S4 **Strain specific CDSs between** ***C. jejuni*** **subsp.** ***jejuni*** **07001,** ***C. jejuni*** **81-176, 81-116, NCTC11168, RM1221 and** ***C. jejuni*** **subsp.** ***doylei*** **269.97.** BLASTN analyses were performed between *C. jejuni* 07001 and five other sequenced genomes of strains: *C. jejuni* subsp. *jejuni* 81-176, 81-116, NCTC11168, RM1221 and *C. jejuni* subsp. *doylei* 269.97. 236, 256, 284, 468, and 544 CDS were not observed in strain 07001 that were specific to strain 81-176, 81-116, NCTC11168, RM1221 and 269.97. The strain specific CDSs in *C. jejuni* 81-176, 81-116, NCTC11168, RM1221 and 269.97 were listed in the Supplementary File S4.
(XLS)

File S5 **Comparisons of the virulence and virulence associated genes between** ***C. jejuni*** **subsp.** ***jejuni*** **07001,** ***C. jejuni*** **81-176, 81-116, NCTC11168, RM1221 and** ***C. jejuni*** **subsp.** ***doylei*** **269.97.** Twenty-seven virulence and virulence associated genes common to Campyl*obacter sp*. were compared between the selected *C. jejuni* strains. The comparison results were summarized in the supplementary File S5.
(XLS)

Table S1 **Genome comparisons between** ***C. jejuni*** **strains.** The background information of the nine respective strains examined in this study is described in the supplementary Table S1.
(DOC)

Table S2 ***Campylobacter*** **plasmid characteristics and homologous ORFs.** Six sequenced *Campylobacter* plasmids were selected for the comparative analysis. The reference ID and the characteristics of the selected plasmids were shown in the supplementary Table S2.
(DOC)

Table S3 **Localization of** ***C. jejuni*** **ICDCCJ07001 homo-polymeric G_C tracts.** The location and the characteristics of the poly (G_C) tracts are described in the supplementary Table S3.
(DOC)

Table S4 **CDS common to Campylobacteriosis outbreak and GBS-associated strains.** *C. jejuni* isolates were classified in the context of disease presentation: Group 1, Group 2 and Group 3. 1,327 genes were common to all groups compared to 27 and 13 unique CDS identified between the Campylobacteriosis outbreak-associated and GBS-associated groups, respectively. CDS corresponding to the different groups are listed in the supplementary Table S4.
(DOC)
