# Supplementary material for: Genomic Characterization of the Guillain-Barre Syndrome-Associated Campylobacter jejuni ICDCCJ07001 Isolate
Source: PLoS One. 2010 Nov 29;5(11):e15060. doi: 10.1371/journal.pone.0015060 (PMC2993937; doi:10.1371/journal.pone.0015060)
Supplement: Table S1 — Genome comparisons between C. jejuni strains. The background information of the nine respective strains examined in this study is described in the supplementary Table S1. (DOC) [file pone.0015060.s006.doc]

**Table S1. Genome comparisons between *C. jejuni* strains**

| **Strains** | **ICDCCJ07001** | **81-176** | **81-116** | **NCTC**  **11168** | **RM1221** | **269.97** | **260.94** | **HB93-13** | **CF93-6** |
| --- | --- | --- | --- | --- | --- | --- | --- | --- | --- |
| **Sub.sps** | *Cjj*^a^ | *Cjj* | *Cjj* | *Cjj* | *Cjj* | *Cjd* | *Cjj* | *Cjj* | *Cjj* |
| **Source** | Human Stool | Human Stool | Human Stool | Human Stool | Chicken Skin | Human Blood | Human Stool | Human Stool | Human Stool |
| **Disease** | GBS  (AMAN) | GI | GI | GI | -^b^ | Bacteremia | GBS (AIDP) | GBS (AMAN) | GBS (MFS) |
| **Epidemic** | Outbreak | Outbreak | Outbreak | Sporadic | Sporadic | Sporadic | Sporadic | Sporadic | Sporadic |
| **Country** | China | USA | UK | UK | USA | South Africa | South Africa | China | Japan |
| **Serotype** | HS:41 | HS:23,36 | HS:6 | HS:2 | HS:53 | - | HS:41 | HS:19 | - |
| **RefSeq ID** | CP002029 | [NC_008787](http://www.ncbi.nlm.nih.gov/sites/entrez?Db=genome&Cmd=ShowDetailView&TermToSearch=20278)  (genome)  NC_008770  (pVir)  [NC_008790](http://www.ncbi.nlm.nih.gov/sites/entrez?Db=genome&Cmd=ShowDetailView&TermToSearch=20281)  (pTet) | [NC_009839](http://www.ncbi.nlm.nih.gov/sites/entrez?Db=genome&Cmd=ShowDetailView&TermToSearch=21421) | [NC_002163](http://www.ncbi.nlm.nih.gov/sites/entrez?Db=genome&Cmd=ShowDetailView&TermToSearch=152) | [NC_003912](http://www.ncbi.nlm.nih.gov/sites/entrez?Db=genome&Cmd=ShowDetailView&TermToSearch=606) | [NC_009707](http://www.ncbi.nlm.nih.gov/sites/entrez?Db=genome&Cmd=ShowDetailView&TermToSearch=21264) | NZ_AANK00000000 | NZ_AANQ00000000  (genome)  NZ_AANQ01000006  (pTet) | NZ_AANJ00000000 |
| **Chromosome Size** | 1.66Mb | 1.62Mb | 1.63Mb | 1.64Mb | 1.78Mb | 1.85Mb | 1.66Mb^c^ | 1.69Mb^c^ | 1.67Mb^c^ |
| **G+C content** | 30.60% | 30.62% | 30.54% | 30.55% | 30.31% | 30.60% | 30.50% | 30.60% | 30.50% |
| **ORF numbers** | 1,579 | 1653 | 1,626 | 1,634 | 1,835 | 1731 | 1,717 | 1,694 | 1,757 |
| **Plasmid** | 1 | 2 | 0 | 0 | 0 | 0 | - | 1 | - |
| **Phage island** | 1 | 0 | 0 | 0 | 4 | - | 1 | - | - |

*^a^C. jejuni* subsp. *jejuni* (*Cjj*)

^b^ -, not determined

^c^Genome incomplete
