# Supplementary material for: Genomic Characterization of the Guillain-Barre Syndrome-Associated Campylobacter jejuni ICDCCJ07001 Isolate
Source: PLoS One. 2010 Nov 29;5(11):e15060. doi: 10.1371/journal.pone.0015060 (PMC2993937; doi:10.1371/journal.pone.0015060)
Supplement: Table S2 — Campylobacter plasmid characteristics and homologous ORFs. Six sequenced Campylobacter plasmids were selected for the comparative analysis. The reference ID and the characteristics of the selected plasmids were shown in the supplementary Table S2. (DOC) [file pone.0015060.s007.doc]

**Table S2. *Campylobacter* plasmid characteristics and homologous ORFs**

| **Plasmid Name** | **ICDCCJ07001_pTet** | **81-176_pTet** | **81-176_pVir** | **HB93-13_pTet** | **pCC31_pTet** | **pCCON31** | **pCCON16** |
| --- | --- | --- | --- | --- | --- | --- | --- |
| **RefSeq ID** | CP002030 | NC_008790 | NC_008770 | NZ_AANQ01000006 | NC_006134 | NC_009795 | NC_009796 |
| **Original strain** | *C. jejuni* ICDCCJ07001 | *C. jejuni* 81-176 | *C. jejuni* 81-176 | *C. jejuni*  HB93-13 | *C. coli* CC31 | *C. concisus* 13826 | *C. concisus* 13826 |
| **Length (bp)** | 44,084 | 45,025 | 37,473 | 38,874 | 44,707 | 30,949 | 16,457 |
| **GC content (%)** | 28.69% | 29.09% | 25.89% | 28.98% | 29.84% | 31.61% | 33.46% |
| **ORF #** | 37 | 52 | 53 | 42 | 50 | 33 | 23 |
| **Gene Density (ORFs/kb)** | 0.84 | 1.15 | 1.41 | 1.08 | 1.12 | 1.07 | 1.4 |
| **Average gene length (bp)** | 968 | 803 | 604 | 768 | 829 | 770 | 637 |
| **Largest ORF Length (bp)** | 5,808 | 5,799 | 2,469 | 2,769 | 5,799 | 5,160 | 1,866 |
| **Homologous ORF** | 37 | 26 | 2 | 21 | 24 | 0 | 0 |
