# Supplementary material for: Genomic Characterization of the Guillain-Barre Syndrome-Associated Campylobacter jejuni ICDCCJ07001 Isolate
Source: PLoS One. 2010 Nov 29;5(11):e15060. doi: 10.1371/journal.pone.0015060 (PMC2993937; doi:10.1371/journal.pone.0015060)
Supplement: Table S3 — Localization of C. jejuni ICDCCJ07001 homo-polymeric G_C tracts. The location and the characteristics of the poly (G_C) tracts are described in the supplementary Table S3. (DOC) [file pone.0015060.s008.doc]

**Table S3. Localization of *C. jejuni* ICDCCJ07001 homo-polymeric** **G_C tracts**

| **No.** | **G_C tracts** | **Position on the Genome** | **Affected CDSs** | **Putative function** | **Characteristics** |
| --- | --- | --- | --- | --- | --- |
| 1 | C (10) | 36,899-36,908 | Intergenic  (ICDCCJ07001_25 -ICDCCJ07001_26) |  | Before the first 16S_rRNA |
| 2 | C (10) | 72,568-72,577 | ICDCCJ07001_49 | putative iron-binding protein | Similar to Cj0045c in NCTC11168 |
| 3 | G (7) | 84,059-84,065 | ICDCCJ07001_62 | chlorohydrolase |  |
| 4 | C (11) | 397,925-397,935 | Intergenic  (ICDCCJ07001_392-ICDCCJ07001_393) |  | Before the second 16S_rRNA |
| 5 | G (7) | 492,974-492,980 | ICDCCJ07001_480 | succinyl-CoA synthase |  |
| 6 | G (10) | 522300-522309 | Intergenic  (ICDCCJ07001_509-ICDCCJ07001_510) |  |  |
| 7 | G (10) | 572,078-572,087 | ICDCCJ07001_566 | conserved hypothetical protein | Similar with Cj0617 in NCTC11168 |
| 8 | G (9) | 631,308-631,316 | ICDCCJ07001_622 | invasion phenotype protein | Similar with Cj0685c in NCTC11168 |
| 9 | G (10) | 721,071-721,080 | ICDCCJ07001_711 | adhesive protein CupB5 |  |
| 10 | C (8) | 724,153-724,160 | Intergenic  ICDCCJ07001_714-ICDCCJ07001_715 |  | Before the third 16S_rRNA |
| 11 | C (9) | 1,026,399-1,026,407 | ICDCCJ07001_1013 | type II restriction-modification enzyme |  |
| 12 | C (9) | 1,275,053-1,275,061 | ICDCCJ07001_1249 | hypothetical protein | FM region in ICDCCJ07001 |
| 13 | G(9) | 1,278,402-1,278,410 | ICDCCJ07001_1253 | formyl transferase domain protein | FM region in ICDCCJ07001 |
| 14 | C(9) | 1,279,488-1,279,496 | ICDCCJ07001_1254 | conserved hypothetical protein | FM region in ICDCCJ07001 |
| 15 | G (8) | 1,281,241-1,281,248 | Intergenic  ICDCCJ07001_1255-ICDCCJ07001_1256 |  | FM region in ICDCCJ07001 |
| 16 | G (9) | 1,288,130-1,288,138 | Intergenic  ICDCCJ07001_1263-ICDCCJ07001_1264 |  | FM region in ICDCCJ07001 |
| 17 | G (9) | 121,956-1,291,964 | ICDCCJ07001_1267 | hypothetical protein | FM region in ICDCCJ07001, similar with Cj1318 in NCTC11168 |
| 18 | C (9) | 1,301,871-1,301,887 | ICDCCJ07001_1274 | hypothetical protein | similar with Cj1342c in NCTC11168 |
| 19 | C (11) | 1,376,455-1,376,465 | Intergenic  ICDCCJ07001_1344-ICDCCJ07001_1345 |  | CPS region in ICDCCJ07001 |
| 20 | C (10) | 1,384,176-1,384,185 | ICDCCJ07001_1351 | sugar transferase | CPS region in ICDCCJ07001, similar with Cj 1420 in NCTC11168 |
| 21 | C (9) | 1,384,352-1,384,360 | ICDCCJ07001_1352 | capsular polysaccharide biosynthesis protein | CPS region in ICDCCJ07001 |
| 22 | C (11) | 1,387,627-1,387,637 | ICDCCJ07001_1355 | putative sugar transferase | CPS region in ICDCCJ07001 |
| 23 | C (9) | 1,391,773-1,391,781 | ICDCCJ07001_1359 | GDP-L-fucose synthase | CPS region in ICDCCJ07001 |
| 24 | C (11) | 1,398,344-1,398,354 | ICDCCJ07001_1365 | CDP-4-dehydro-6-deoxy-D-glucose 3-epimerase | CPS region in ICDCCJ07001 |
| 25 | C (11) | 1,634,467-1,634,477 | ICDCCJ07001_1596 | hypothetical protein |  |
