# Supplementary material for: Genomic Characterization of the Guillain-Barre Syndrome-Associated Campylobacter jejuni ICDCCJ07001 Isolate
Source: PLoS One. 2010 Nov 29;5(11):e15060. doi: 10.1371/journal.pone.0015060 (PMC2993937; doi:10.1371/journal.pone.0015060)
Supplement: Table S4 — CDS common to Campylobacteriosis outbreak and GBS-associated strains. C. jejuni isolates were classified in the context of disease presentation: Group 1, Group 2 and Group 3. 1,327 genes were common to all groups compared to 27 and 13 unique CDS identified between the Campylobacteriosis outbreak-associated and GBS-associated groups, respectively. CDS corresponding to the different groups are listed in the supplementary Table S4. (DOC) [file pone.0015060.s009.doc]

**Table S4. CDS common to Campylobacteriosis outbreak and GBS-associated strains**

| **Campylobacteriosis Outbreak-Associated group** | | |
| --- | --- | --- |
| **CDS name** | **Length** | **NCBI NR Annotation** |
| ICDCCJ07001_31 | 1971 | conserved hypothetical protein |
| ICDCCJ07001_32 | 2070 | membrane protein, putative |
| ICDCCJ07001_33 | 585 | conserved hypothetical protein |
| ICDCCJ07001_34 | 2706 | cytochrome c biogenesis protein |
| ICDCCJ07001_35 | 1671 | hypothetical protein C8J_0033 |
| ICDCCJ07001_204 | 576 | hypothetical protein C8J_0198 |
| ICDCCJ07001_205 | 1938 | serine protease eatA precursor |
| ICDCCJ07001_510 | 744 | conserved hypothetical protein |
| ICDCCJ07001_511 | 867 | conserved hypothetical protein |
| ICDCCJ07001_512 | 732 | conserved hypothetical protein |
| ICDCCJ07001_513 | 465 | hypothetical protein |
| ICDCCJ07001_514 | 306 | conserved hypothetical protein |
| ICDCCJ07001_613 | 1341 | potassium-transporting ATPase |
| ICDCCJ07001_737 | 1227 | histidyl-tRNA synthetase |
| ICDCCJ07001_788 | 771 | amino acid-binding protein |
| ICDCCJ07001_830 | 1770 | arylsulfate sulfotransferase |
| ICDCCJ07001_890 | 360 | conserved hypothetical protein |
| ICDCCJ07001_891 | 885 | conserved hypothetical protein |
| ICDCCJ07001_892 | 525 | conserved hypothetical protein |
| ICDCCJ07001_893 | 957 | conserved hypothetical protein |
| ICDCCJ07001_1115 | 522 | conserved hypothetical protein |
| ICDCCJ07001_1253 | 720 | formyl transferase domain protein |
| ICDCCJ07001_1268 | 864 | protein of unknown function |
| ICDCCJ07001_1269 | 474 | protein of unknown function |
| ICDCCJ07001_1273 | 1827 | motility accessory factor |
| ICDCCJ07001_1303 | 1065 | radical SAM domain protein |
| ICDCCJ07001_1626 | 378 | possible outer membrane protein |
| **GBS-Associated Group** | | |
| ICDCCJ07001_65 | 609 | Hemerythrin HHE cation binding domain subfamily |
| ICDCCJ07001_696 | 855 | type II DNA modification methyltransferase, putative |
| ICDCCJ07001_698 | 630 | phage repressor protein |
| ICDCCJ07001_1447 | 1455 | cryptic C4-dicarboxylate transporter DcuD |
| ICDCCJ07001_1093 | 1257 | lipooligosaccharide biosynthesis glycosyltransferase |
| ICDCCJ07001_1094 | 1170 | putative glycosyl transferase |
| ICDCCJ07001_1095 | 555 | beta-1,4-N-acetylgalactosaminyltransferase |
| ICDCCJ07001_1096 | 351 | beta-1,4-N-acetylgalactosaminyltransferase |
| ICDCCJ07001_1097 | 906 | beta-1,3-galactosyltransferase |
| ICDCCJ07001_1098 | 876 | alpha-2,3-/2,8-sialyltransferase |
| ICDCCJ07001_1101 | 666 | N-acylneuraminate cytidylyltransferase |
| ICDCCJ07001_1102 | 447 | acetyltransferase |
| ICDCCJ07001_1103 | 342 | acetyltransferase |
